# Fine-tuning germline mutation rates across evolution

**DOI:** 10.1016/j.tig.2023.05.001

**Published:** 2023-08

**Authors:** Stephen J. Bush, Anne Goriely

**Affiliations:** 1 MRC Weatherall Institute of Molecular Medicine, Radcliffe Department of Medicine, University of Oxford, Oxford, UK

## Abstract

The germline mutation rate (GMR) sets the pace at which mutations, the raw material of evolution, are introduced into the genome. By sequencing a dataset of unprecedently broad phylogenetic scope, Bergeron *et al**.* estimated species-specific GMR, offering numerous insights into how this parameter shapes and is shaped by life-history traits.

Without new DNA variations, there can be no innovation, no adaptation, no evolution, and (in short) no long-term survival for a species. How, why, and how often new mutations arise and are introduced into the genome are fundamental questions. Traditionally, the germline mutation rate (GMR) has been estimated using phylogenetic methods but more recently it has become possible to assess the GMR directly, using pedigrees to sequence parent–child trios [1] (Figure 1). Although both approaches have merit, they measure different things on different time-frames. Phylogenetic methods assess divergence on the ‘long-scale’, using external calibration points (such as the fossil record) and substitution rates between species. Trio sequencing operates at the other extreme, on the ‘short-scale’ of a single generation, capturing newly arising variations in individual genomes, so-called *de novo* mutations (DNMs). Importantly, the trio approach also allows us to probe why and how DNMs occur in the first place. For example, we now know that, in humans, the majority (~80%) of DNMs originate in the male germline and that their number increases with paternal age. This suggests that the way gametogenesis is controlled is a key regulator of GMR [2].

**Figure 1:**
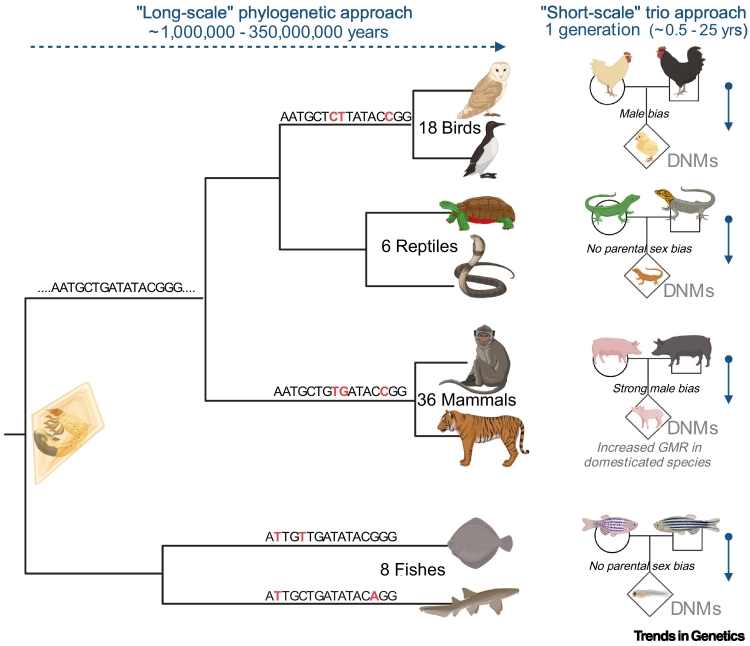
Estimating the germline mutation rate (GMR) across and within different species. GMRs can be measured on both the ‘long-scale’ (horizontal arrow) and ‘short-scale’ (vertical arrows) time-frames using phylogenetic and trio-sequencing approaches, respectively. Phylogenetic approaches require external calibration points (the amber fossil on the left) and quantification of substitutions between species (the red bases in the short consensus DNA sequence are shown as a representative illustration), whereas trio-sequencing quantifies the number of *de novo* mutations (DNMs) as they arise in a single generation. DNMs are DNA sequence variations found in the child that are not present in either parents’ DNA and have likely occurred during spermatogenesis or oogenesis. The phylogenetic tree shows an overview of the scope (with the four main vertebrate classes) and sample size of Bergeron *et al.* [3], with several conclusions of the work (regarding parental sex biases in DNM origin) indicated on a few representative trios. Figure created with BioRender.com.

Reconciling the two approaches, Bergeron *et al.* [3] published a breakthrough study on GMR, producing a trio-sequencing dataset of considerable phylogenetic scope. By sequencing 151 parent–child trios from 68 different vertebrates, they charted the range of species-specific GMRs as they arose across one generation and identified a 40-fold variation across all species. However, all things considered, the variation in GMR within each of four major vertebrate classes (mammals, birds, reptiles, and fish) was ‘arguably modest’, which is rather surprising. For example, despite their divergent evolutionary histories and living environments, the GMRs of humans and penguins were shown to be quite similar.

To understand GMR variation across species, the authors considered differences in male versus. female reproductive strategies. A proportion of DNMs could be phased (i.e., assigned to their parental chromosome of origin), which revealed a pronounced paternal bias in DNM origin in every mammal and bird studied. In these species, oogenesis differs from spermatogenesis: the latter is typically maintained by actively dividing stem cells supporting abundant sperm production but leading to the accumulation of copy-errors during genome replication [2]. Consistently, the male bias was not as pronounced in reptiles or fish, which often produce a much larger number of eggs or have developed other adaptations to mitigate the bias, such as male seasonal breeding.

Complementing this, significant positive associations were also found between GMR and several life-history traits related to reproduction: generation time, maturation time, and fecundity. A striking observation was a higher mutation rate in domesticated species, likely the consequence of recent artificial selection for desirable traits, such as increased fecundity or early sexual maturation. Similarly, GMR was negatively associated with effective population size (*N*_e_, the number of breeding individuals contributing to the next generation). This lends further support to the ‘drift barrier’ hypothesis of mutation rate evolution [4], whereby variation in *N*_e_ affects the strength of selection relative to genetic drift and thereby the likelihood that mutations will be fixed or purged in the population.

Taken together, this suggests that GMR is a parameter that has been finely-tuned by both natural selection and neutral evolutionary forces, likely because the incidence of germline mutations has a discernible effect upon fitness. In humans, for example, severe developmental disorders caused by a DNM occur in ~1 in 300 births [5]. These observations raise important, and as-yet-unanswered, questions about the mechanisms by which GMRs are controlled in different species and the extent to which these may be shared by somatic cells or are unique to the germline.

A recent study performed a comparative evaluation of multiple cell types from the same (human) individual, allowing an unbiased comparison of the mutational landscape in the soma and the germline [6]. Compared with the somatic mutation rate (which is itself evolutionarily constrained, negatively scaling with lifespan [7]), the GMR was significantly lower. While this may not be surprising, it will be important to shed light on the mechanisms by which GMRs are kept in check and establish if it is also the case in other species. Although hypotheses such as ‘transcriptional scanning’ (i.e., the idea that widespread germline transcription can reduce mutagenesis through transcription-coupled repair [8]) have been put forward, they have not garnered much evidential support, suggesting that other factors intrinsic to germline maintenance are likely at work.

Owing to advances in single-cell sequencing, multi-species comparative analyses of the molecular evolution of the germline have recently been possible [9] and in broadening the phylogenetic scope of enquiry, Bergeron *et al.* draw further attention to this area. The spermatogonia, the male germline stem cells, have to balance the contradictory demands of regular mitotic proliferation, which is inherently mutagenic, to ensure an organism’s fertility, while limiting the introduction of deleterious mutations in order to maintain genome integrity across generations. This interplay of forces is of fundamental importance, shaping and being shaped by life history and manifesting as a finely calibrated GMR. Reproductive traits can vary greatly even among closely related species (e.g., chimp testes are three to ten times larger than those of humans), yet their GMRs can be similar.

Given the challenging nature of sample collection, the authors note some unavoidable limitations to their study, including the fact that some species were only represented by a single family trio, for which the ages of the parents, a key determinant of DNM number, may not be representative of those in wild populations. Because DNMs are rare events, accurate GMRs need to be obtained genome-wide, which necessitate access to (near-) complete reference genomes, but many have not yet been assembled to chromosome level. The development of higher-quality reference resources for non-model species [by, e.g., the Darwin Tree of Life (www.darwintreeoflife.org) or the Zoonomia consortium] [10] is an ongoing community effort.

The combined efforts of these pioneering studies should inspire multiple directions for future research; as such, it is only a matter of time before the long- and short-scale approaches to estimating GMRs and the selective forces shaping genomes are further refined, compared and, eventually, reconciled.

## Declaration of interests

The authors declare no conflicts of interest.

## References

[1] Campbell C.R. (2021). Pedigree-based and phylogenetic methods support surprising patterns of mutation rate and spectrum in the gray mouse lemur. Heredity.

[2] Wood K.A., Goriely A. (2022). The impact of paternal age on new mutations and disease in the next generation. Fertil. Steril..

[3] Bergeron L.A. (2023). Evolution of the germline mutation rate across vertebrates. Nature.

[4] Sung W. (2012). Drift-barrier hypothesis and mutation-rate evolution. Proc. Natl. Acad. Sci. U. S. A..

[5] McRae J.F. (2017). Prevalence and architecture of de novo mutations in developmental disorders. Nature.

[6] Moore L. (2021). The mutational landscape of human somatic and germline cells. Nature.

[7] Cagan A. (2022). Somatic mutation rates scale with lifespan across mammals. Nature.

[8] Xia B. (2020). Widespread transcriptional scanning in the testis modulates gene evolution rates. Cell.

[9] Murat F. (2023). The molecular evolution of spermatogenesis across mammals. Nature.

[10] Christmas M.J. (2023). Evolutionary constraint and innovation across hundreds of placental mammals. Science.

